# Supplementary material for: Sorting at embryonic boundaries requires high heterotypic interfacial tension
Source: Nat Commun. 2017 Jul 31;8:157. doi: 10.1038/s41467-017-00146-x (PMC5537356; doi:10.1038/s41467-017-00146-x)
Supplement: Supplementary file 2 — Supplementary Software 1 [file 41467_2017_146_MOESM2_ESM.zip › PottsModel/SrcPottsModel/doc/model/Constants.GuiManager.html]

Constants.GuiManager


---


|  |  |  |
| --- | --- | --- |
| **Overview** \| **Package** \| **Class** \| **Use** \| **Tree** \| **Deprecated** \| **Index** \| **Help** | |  |
| **PREV CLASS**   **NEXT CLASS** | **FRAMES**    **NO FRAMES**     **All Classes** |  |
| SUMMARY: NESTED \| FIELD \| CONSTR \| METHOD | DETAIL: FIELD \| CONSTR \| METHOD |  |


---


## model Class Constants.GuiManager

```
java.lang.Object
  model.Constants.Manager
      model.Constants.GuiManager
```

**Enclosing class:**: Constants

---

``` private class Constants.GuiManager extends Constants.Manager ```

Manages graphical output information related to this simulation.

---

| **Field Summary** | |
| --- | --- |
| `private  boolean` | `enabled` |
| `private  int` | `pixelSize` |
| `private  boolean` | `scalePixelSize` |
| `private  int` | `screenHeight` |
| `private  int` | `screenWidth` |

**Fields inherited from class model.Constants.Manager**

`prop`


**Constructor Summary**

`Constants.GuiManager(java.util.Properties p)`


| **Method Summary** | |
| --- | --- |
| `(package private)  void` | `processProperties()` |

**Methods inherited from class java.lang.Object**

`clone, equals, finalize, getClass, hashCode, notify, notifyAll, toString, wait, wait, wait`

**Field Detail**

### enabled

```
private boolean enabled
```

---


### pixelSize

```
private int pixelSize
```

---


### scalePixelSize

```
private boolean scalePixelSize
```

---


### screenWidth

```
private int screenWidth
```

---


### screenHeight

```
private int screenHeight
```


**Constructor Detail**

### Constants.GuiManager

```
public Constants.GuiManager(java.util.Properties p)
```


**Method Detail**

### processProperties

```
void processProperties()
```

:   **Specified by:**: `processProperties` in class `Constants.Manager`


---


|  |  |  |
| --- | --- | --- |
| **Overview** \| **Package** \| **Class** \| **Use** \| **Tree** \| **Deprecated** \| **Index** \| **Help** | |  |
| **PREV CLASS**   **NEXT CLASS** | **FRAMES**    **NO FRAMES**     **All Classes** |  |
| SUMMARY: NESTED \| FIELD \| CONSTR \| METHOD | DETAIL: FIELD \| CONSTR \| METHOD |  |


---
